# ABCA1, apoA-I, and BTN3A1: A Legitimate Ménage à Trois in Dendritic Cells

**DOI:** 10.3389/fimmu.2018.01246

**Published:** 2018-06-08

**Authors:** Chiara Riganti, Barbara Castella, Massimo Massaia

**Affiliations:** ^1^Dipartimento di Oncologia, Università degli Studi di Torino, Turin, Italy; ^2^Laboratorio di Immunologia dei Tumori del Sangue (LITS), Centro Interdipartimentale di Ricerca in Biologia Molecolare (CIRBM), Università degli Studi di Torino, Turin, Italy; ^3^SC Ematologia, AO S. Croce e Carle, Cuneo, Italy

**Keywords:** Vγ9Vδ2 T cells, phosphoantigens, isopentenyl pyrophosphate, ATP-binding cassette transporter A1, apolipoprotein A-I, butyrophilin-3A1

## Abstract

Human Vγ9Vδ2 T cells have the capacity to detect supra-physiological concentrations of phosphoantigens (pAgs) generated by the mevalonate (Mev) pathway of mammalian cells under specific circumstances. Isopentenyl pyrophosphate (IPP) is the prototypic pAg recognized by Vγ9Vδ2 T cells. B-cell derived tumor cells (i.e., lymphoma and myeloma cells) and dendritic cells (DCs) are privileged targets of Vγ9Vδ2 T cells because they generate significant amounts of IPP which can be boosted with zoledronic acid (ZA). ZA is the most potent aminobisphosphonate (NBP) clinically available to inhibit osteoclast activation and a very potent inhibitor of farnesyl pyrophosphate synthase in the Mev pathway. ZA-treated DCs generate and release in the supernatants picomolar IPP concentrations which are sufficient to induce the activation of Vγ9Vδ2 T cells. We have recently shown that the ATP-binding cassette transporter A1 (ABCA1) plays a major role in the extracellular release of IPP from ZA-treated DCs. This novel ABCA1 function is fine-tuned by physical interactions with IPP, apolipoprotein A-I (apoA-I), and butyrophilin-3A1 (BTN3A1). The mechanisms by which soluble IPP induces Vγ9Vδ2 T-cell activation remain to be elucidated. It is possible that soluble IPP binds to BTN3A1, apoA-I, or other unknown molecules on the cell surface of bystander cells like monocytes, NK cells, Vγ9Vδ2 T cells, or any other cell locally present. Investigating this scenario may represent a unique opportunity to further characterize the role of BTN3A1 and other molecules in the recognition of soluble IPP by Vγ9Vδ2 T cells.

## Introduction

A very peculiar feature of Vγ9Vδ2 T cells is their TCR-dependent, MHC-independent recognition of phosphoantigens (pAgs) (1). pAgs are pyrophosphorylated isoprenoids generated in the mevalonate (Mev) pathway of mammalian cells. Isopentenyl pyrophosphate (IPP) is the prototypic pAg recognized by Vγ9Vδ2 T cells (2). Increased Mev pathway dysregulation has been reported in many types of cancer cells (3). This metabolic derangement leads to increased IPP production which is sensed by Vγ9Vδ2 T cells laying the basis of their multifaceted contribution to immune surveillance and antitumor immunity (4).

Vγ9Vδ2 T cells also recognize pAgs generated in Mev and non-Mev pathway of microbial pathogens [i.e., hydroxyl dimethylallyl pyrophosphate (HDMAPP), hydroxy-methyl-butyl-pyrophosphate (HMBPP)] (5, 6); this capacity confers to Vγ9Vδ2 T cells a critical role in innate and adaptive antimicrobial immune responses (7).

The third pAg category recognized by Vγ9Vδ2 T cells are the synthetic pAgs developed for therapeutic purposes [i.e., bromohydrinpyrophosphate, (2E)-1-hydroxy-2-methylpent-2-enyl pyrophosphate (CHDMAPP)] (8, 9). Some of these compounds have been investigated in clinical trials with alternating success (10) and are currently used as research tools to directly or indirectly activate Vγ9Vδ2 T cells *in vitro* (11–13). More recently, several technologies have been used to generate pAg prodrugs with the aim to overcome the poor cell membrane permeability and limited *in vivo* stability of pyrophosphate containing pAgs (14, 15).

Another strategy which has been used *in vivo* and *in vitro* to activate Vγ9Vδ2 T cells is to intentionally increase intracellular IPP concentrations in tumor cells and/or antigen-presenting cells (APCs) like monocytes or dendritic cells (DCs) with aminobisphosphonates (NBP) (16), and alkylamines (17, 18). These compounds inhibit farnesylpyrophosphate synthase (FPPS) in the Mev pathway causing intracellular IPP accumulation (18–20). Prodrug technology has also been used to develop an highly hydrophobic NBP prodrug [tetrakis-pivaloyloxymethyl 2-(thiazole-2-ylamino) ethylidene-1,1-bisphosphonate (PTA)] to facilitate intracellular uptake and, after conversion into the active form, to induce FPPS blockade and IPP accumulation (21).

The fate of supra-physiological IPP concentrations is different according to cell type and tissue localization. Intracellular formation of the pro-apoptotic ATP analog 1-adenosin-5-yl 3-(3-methylbut-3-enyl) triphosphoric acid diester (ApppI) formation depends on the activity of FPPS, aminoacyl-tRNA synthetases, dosage, and potency of NBP (22). Zoledronic acid (ZA), the most potent NBP clinically available, is commonly used to treat bone disease in myeloma and solid cancers with bone metastases (23–25). In osteoclasts, ZA-induced supra-physiological IPP concentrations leads to intracellular ApppI formation (26). ApppI initiates the apoptotic program in osteoclasts explaining the therapeutic efficacy of ZA in this setting. Tumor cells also accumulate intracellular apoptotic ApppI concentrations when exposed to ZA concentrations similar to those achieved in the mineralized bone (from 50 µM to 1 mM). Much lower ZA concentrations (0.5–1 µM) are used to boost the capacity of tumor cells, monocytes, and DCs to activate Vγ9Vδ2 T cells (19, 27, 28). Under these conditions, ZA-induced IPP accumulation is insufficient to induce enough ApppI to trigger apoptosis. It is highly conceivable that APCs like monocytes and DCs have developed mechanisms to resist the toxic effects of intracellular IPP accumulation and converted this resilience to survive and recruit Vγ9Vδ2 T cells. Upregulation of IPP extruders like ABCA1 could contribute to this resilience (see also below).

Zoledronic acid-treated mature DCs are better Vγ9Vδ2 T-cell activators than ZA-treated monocytes or ZA-treated immature DCs (29). This superiority is directly related to their capacity to accumulate high intracellular IPP concentrations and to release IPP in the supernatants (SNs) at concentrations up to 1,000× higher (nanomolar range) than intracellular concentrations (picomolar range) (29, 30). These extracellular IPP concentrations are sufficient to induce Vγ9Vδ2 T-cell proliferation in the absence of cell-to-cell contact with ZA-treated DCs (30, 31). How IPP is released in the extracellular microenvironment and delivered to Vγ9Vδ2 T cells has been a matter of investigation and partially decoded over the last year (31). This review is aimed at discussing the role played by ABCA1, apo-AI, and BTN3A1 in the extracellular IPP release from ZA-treated DCs.

## Looking for Membrane-Associated pAg Transporters

F1-ecto-ATPase has been the first cell surface protein associated with IPP presentation to Vγ9Vδ2 T cells. Interest was driven by the discovery that apoA-I and F1-ecto-ATPase discriminate between Vγ9Vδ2 T-cell sensitive or insensitive tumor cell lines (32). The association between IPP and F1-eco-ATPase was reported a few years later in 721.221 B cells (33). This B-cell line is unable to activate Vγ9Vδ2 T cells, unless incubated with high-dose ZA to induce apoptosis. ZA stimulation induces intracellular IPP accumulation, ApppI formation and binding to F1-ecto-ATPase. Allosteric F1-ecto-ATPase modification induced by ApppI leads to Vγ9Vδ2 T-cell activation *via* TCR-dependent recognition (33).

Although very attractive, this model left the field open to several questions. IPP does not directly bind to F1-ecto-ATPase, but it requires ApppI formation; a nucleotide pyrophosphatase (NPP) is then required to release IPP from ApppI and make it available to Vγ9Vδ2 T cells. It is currently unknown whether NPP activity is provided *in cis* by the same cells which have accumulated IPP or *in trans* by neighboring cells. Thus, the IPP/ApppI/F1-ecto-ATPase pathway appears to work as a multistep process in which IPP is initially transformed into ApppI which is relocated to the plasma membrane bound to F1-ecto-ATPase. Next, IPP is made available to bystander Vγ9Vδ2 T cells by NPP which releases IPP from ApppI. Vγ9Vδ2 T cells themselves have been reported to express CD39 ecto-ATPase after activation, but with the opposite goal, i.e., to destroy locally available IPP and downregulate their activation (34). Another issue is that ApppI is mainly generated in apoptotic cells, whereas Vγ9Vδ2 T cells are also activated by non-apoptotic cells (35–37). Finally, HMBPP, HDMAPP, and all HDMAPP-adenylated, thymidylated, and uridylated pyrophosphoric derivatives are potent Vγ9Vδ2 T-cell activators without any capacity to bind F1-ecto-ATPase (9, 38). These nucleotides are released in the extracellular microenvironment by non-apoptotic cells or bacteria and cleaved by extracellular pyrophosphatase (39).

A major advance has been the discovery that F1-ecto-ATPase is a receptor for apolipoprotein A-I (apoA-I) (32, 39) and that apoA-I is necessary for Vγ9Vδ2 T-cell activation by tumor cells expressing IPP/ApppI-loaded F1-ecto-ATPase (32). Since it is very unlikely that F1-ecto-ATPase is released from the plasma membrane, it has been hypothesized that soluble apoA-I may activate Vγ9Vδ2 T cells remotely. Interestingly, chronic inflammation is associated with reduced levels of circulating apoA-I and lower immune competence of Vγ9Vδ2 T cells (40). All these findings have enforced the idea that apoA-I is a necessary player in the efflux, delivery and pAg presentation to Vγ9Vδ2 T cells (32).

## Looking for Soluble pAg Transporters

ApoA-I is physiologically involved in the assembly of nascent high-density lipoproteins (HDL), which mediate the reverse cholesterol transport. The first step in this process is the interaction of apoA-I with the extracellular domain of the ATP-binding cassette transporter A1 (ABCA1), a member of the ABC transmembrane transporter family, abundant in liver, gastrointestinal tract, and macrophages (41). Cholesterol and phospholipids are physiologically effluxed by ABCA1 and loaded by apoA-I, but they are not the only lipids handled by this pathway; α-tocopherol (42), dolichol, and retinoic acid are also effluxed by ABCA1 and transported by apoA-I to nascent HDL (43, 44). Interestingly, all these molecules share multiple isoprenoid moieties identical to that contained in IPP and other Vγ9Vδ2 T-cell activating pAgs.

This structural similarity prompted us to investigate whether the ABCA1/apoA-I system could also extrude intracellular IPP, especially when potentially harmful intracellular concentrations are reached. ZA-treated DCs turned out to be a very convenient and highly reproducible tool to investigate this issue. We have found that ABCA1 plays a major role in the extracellular IPP release from ZA-treated DCs and other cells, and that IPP cannot be released in the SNs of ZA-treated DCs if ABCA1 is not present or functionally active.

So far, we cannot exclude that other isoprenoids structurally related to IPP, like dimethylallyl pyrophosphate, geranyl pyrophosphate, FPP, or geranylgeranyl pyrophosphate (GGPP), are also effluxed by the ABCA1/apoA-I system in DCs and/or other cells. These isoprenoids can also activate Vγ9Vδ2 T cells (45, 46) and regulate the cross-talk between immune cells, cancer cells, and bystander cells in the tumor microenvironment (TME) (47, 48). To exert their mitogenic or regulatory functions in the TME, these metabolites must reach adequate intracellular concentrations to be released in replace of cholesterol and/or phospholipids that are the privileged molecules conveyed by ABCA1/apoA-I. We have shown that IPP extracellular release by ABCA1 overcomes that of cholesterol only when supra-physiological concentration of IPP are reached as a consequence of ZA-induced FPPS inhibitions (31). It is possible that ABCA1 takes the lead in extruding alternative pAgs like HMBPP only when supra-physiological concentrations are reached as reported in neutrophils after internalization of HMBPP-producing bacteria (49). Structure–activity relation studies, cross-linking of radiolabeled pAgs different from IPP should help to clarify this unexplored and exciting issue.

**Figure 1:**
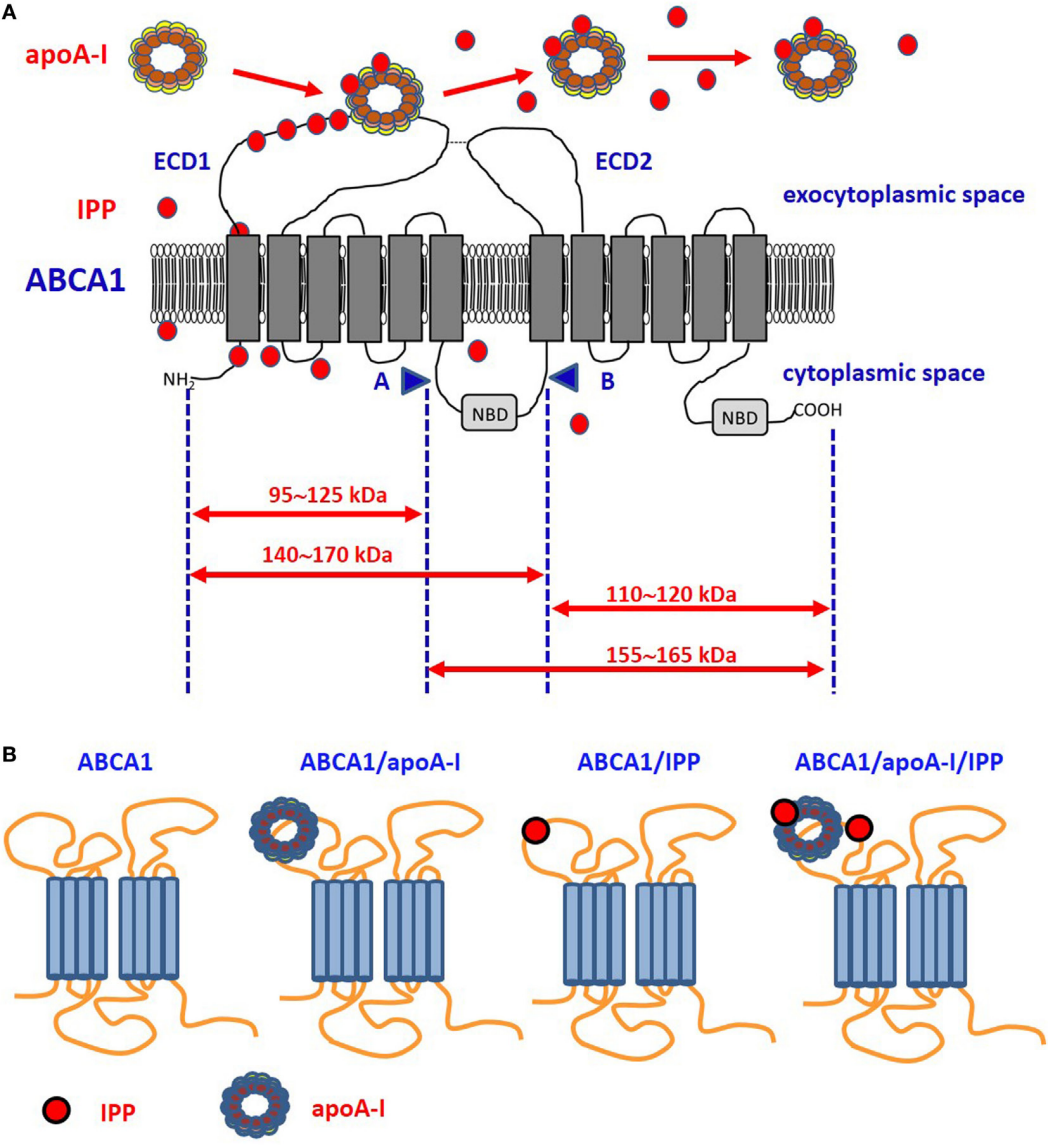
Proposed model of ABCA1, apoA-I, and isopentenyl pyrophosphate (IPP) interactions**. (A)** It is unknown whether intracellular IPP binds to intracellular ABCA1 domains as it does with the intracellular B30.2 domain of BTN3A1 (see Figure 2). IPP is extruded across ABCA1 pore and reaches the extracellular environment. Limited trypsin-digestion cleaves ABCA1 into four fragments, corresponding to different extracellular and intracellular domains (51). The schematic diagram of trypsin-limited digestion of ABCA1 and the molecular sizes of the fragments produced are shown. We have found that IPP is associated with the amino-terminal extracellular portion of ABCA1 (31). Interestingly, apoA-I has been reported to interact with the same portion (52). We propose that IPP and apoA-I meet and associate within the amino-terminal portion of ABCA1 in the ECD1. IPP locally competes with cholesterol and other phospholipids for apoA-I binding and transportation. Local concentrations and the oxidized status of apoA-I, especially if IPP-producing cells are embedded in an inflammatory microenvironment, may favor IPP binding vs other metabolites. It is also possible that IPP is released in the extracellular space unbound to apoA-I. It is currently unknown whether IPP/apoA-I is more resistant to degradation by serum nucleotide pyrophosphatases than soluble IPP and more effective in the activation of Vγ9Vδ2 T cells faraway from IPP-producing cells. **(B)** ABCA1 is schematically represented from left to the right without any extra-loaded molecule, loaded with apoA-I only, with IPP only, and with IPP/apoA-I + IPP. It has been shown that apoA-I and IPP can bind to ABCA1, and that ABCA1 can bind to BTN3A1 (31). It is currently unknown whether ABCA1 has different affinity for BTN3A1 depending on IPP and/or apoA-I loading. Arrows: moleculare weight of fragments derived from trypsin-cleavage sites; COOH, carboxyterminal domain; ECD1, extracellular domain 1; ECD2, extracellular domain 2; NBD, nucleotide binding domain; NH2, amino-terminal domain.

Interestingly, single-nucleotide polymorphisms and posttranslational modifications (i.e., methionine oxidation) reduce apoA-I affinity for cholesterol and increase the affinity for other lipids (50). Since oxidation commonly occurs in the inflammatory microenvironment, it is possible that oxidized apoA-I behave more efficiently as pAg carrier and provide adequate pAg concentrations in inflamed tissues to induce the activation of Vγ9Vδ2 T cells (Figure 1).

## BTN3A1: A Key Player in Vγ9Vδ2 T-Cell Responses to pAg

**Figure 2:**
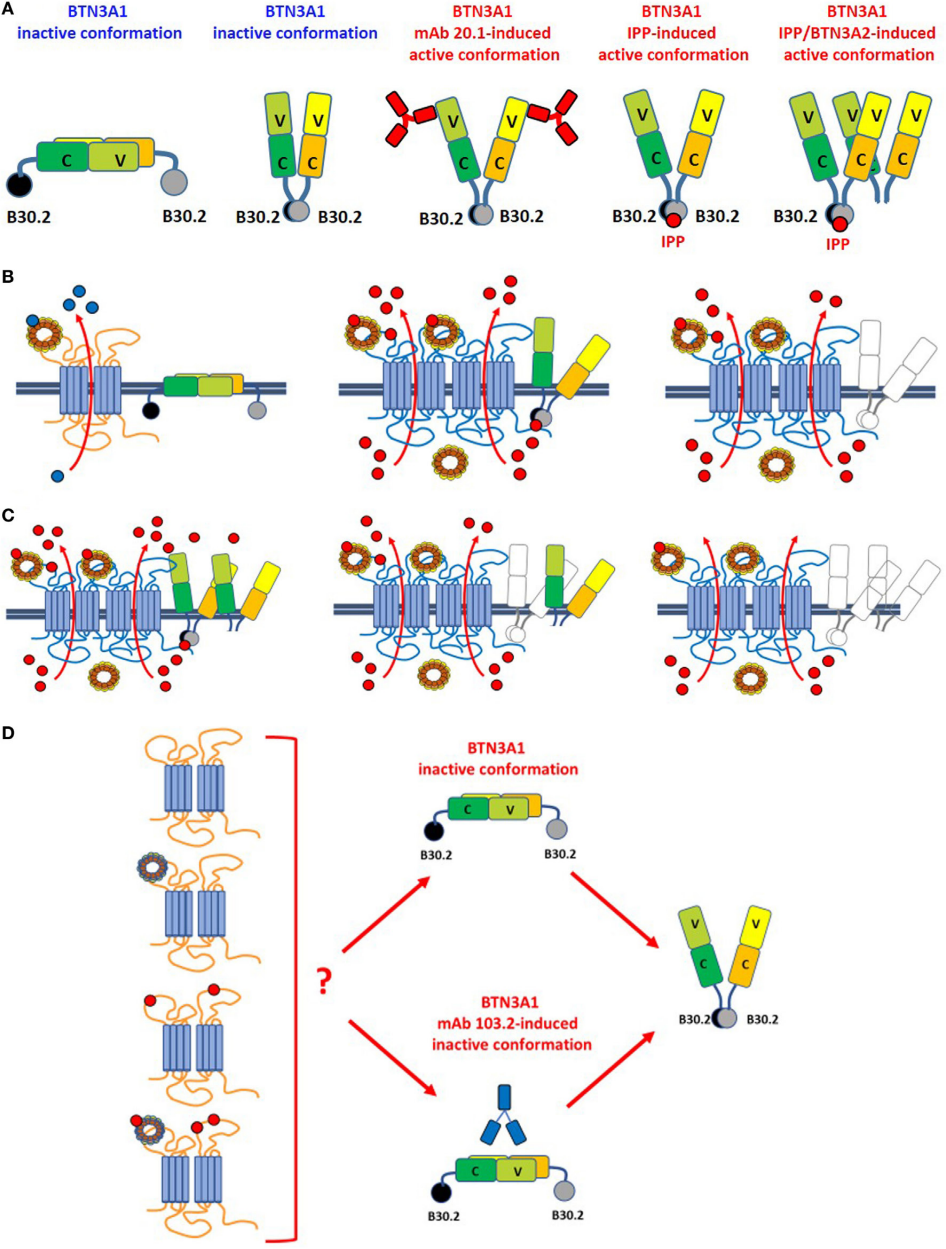
Leads of the ménage à trois between ABCA1, apoA-I, and BTN3A1. **(A)** BTN3A1 inactive and active dimer conformations are represented from left to the right. Models are derived from crystallographic-based models using recombinant proteins and/or immobilized Vγ9Vδ2 TCRs, and tested using Fluorescence Resonance Energy Transfer-based measurements or proximity-ligation assays. These models are inspiring but still unproved in living cells under physiological or pathological conditions. The inactive conformations include both the head-to-tail conformation (left) and a V-shaped conformation (right) (65). Active conformations are characterized by loss of the head-to-tail conformation or by a rotational shift in the V-shaped dimer induced by the agonistic 20.1 mAb (which binds to extracellular IGHV-like domains), isopentenyl pyrophosphate (IPP) (which binds to the intracellular B30.2 domain), or by BTN3A1/BTN3A2 interactions as recently reported by Vantourout et al. (56). **(B)** Hypothetical configuration of ABCA1/apoA-I/BTN3A1 interactions are represented from left to right. *Left*: in the absence of zoledronic acid (ZA)-induced supra-physiological IPP concentrations, BTN3A1 maintains the inactive dimer conformation (for simplicity only the head-to-tail dimer is shown); ABCA1 in cooperation with apoA-I is mainly committed to extrude cholesterol (blue dots). ABCA1 and BTN3A1 are not physically associated under these conditions. *Middle*: hypothetical configuration of ABCA1/apoA-I/BTN3A1 interactions driven by ZA-induced intracellular IPP accumulation (red dots). ABCA1 and apoA-I expressions are increased, BTN3A1 expression is not increased, but BTN3A1 acquires the active dimer conformation because IPP is bound to the intracellular B30.2 domain; the mutually supportive cooperation between ABCA1, apoA-I, and active BTN3A1 leads to the release of picomolar IPP amounts in the extracellular fluids. Whether extracellular IPP (red dots) binds to extracellular IGHV-like domain of BTN3A1 (as shown) and participate to the activation of Vγ9Vδ2 T cells according to the antigen-presentation presentation model proposed by Vavassori (57) is unknown; *Right*: hypothetical scenario in *Btn3a1*-silenced ZA-treated dendritic cells. Desertion of BTN3A1 from the ménage à trois decreases the efficiency of extracellular IPP release by ABCA1/apoA-I even if they remain upregulated (31). These data indicate that the expression of BTN3A1 is useful but dispensable and that the main role is played by ABCA1/apoA-I; **(C)** hypothetical models of ABCA1/apoA-I/BTN3A1-BTN3A2 interactions are represented from left to right. Active BTN3A1 conformation is induced by interactions between BTN3A1/BTN3A2 and IPP bound to the B30.2 domains of BTN3A1. No data are currently available to support the hypothesis that BTN3A2 is physically bound to ABCA1. *Left*: ABCA1 and apoA-I expressions are increased, and BTN3A1 acquires the active dimer conformation because of BTN3A1/BTN3A2/IPP interactions. It is unknown whether this is the most effective complex to extrude IPP. *Middle*: in the experiments reported in Ref. (31), we have silenced BTN3A1 expression only and we know that this complex is still able to release IPP although with a lower efficiency [see also **(B)**, right panel]. One possible explanation is that BTN3A2 partially substitutes for BTN3A1. *Right*: it is conceivable, although yet unproved, that desertion of both BTN3A1 and BTN3A2 from the complex compromises even more extracellular IPP release. **(D)** It is currently unknown whether unloaded ABCA1 or IPP/apoA-I-loaded ABCA1 can switch BTN3A1 from its inactive conformation to the active dimer conformation in the absence of IPP bound to the intracellular B30.2 domain. For simplicity only the head-to-tail dimer is shown. It is also unknown whether unloaded or IPP/apoA-I-loaded ABCA1 can overcome the inactive BTN3A1 conformation locked by the antagonist 103.2 mAb.

One major advance in understanding pAg-induced Vγ9Vδ2 T-cell activation has been the identification of the butyrophilin-3 (BTN3) protein family as a key mediator in this process (53). BTN3 proteins, also known as CD277, are type I transmembrane proteins with two immunoglobulin (Ig)-like extracellular domains (IgV and IgC) and close structural homology with the B7-superfamily of proteins (54, 55). Three isoforms of BTN3A are present in humans: BTN3A1, BTN3A2, and BTN3A3, each encoded by a separate gene. BTN3A1 and BTN3A3 both contain the intracellular B30.2 domains, but BTN3A1 only has the capacity to induce pAg-dependent Vγ9Vδ2 T-cell activation. Recent findings from Vantourout et al. (56) indicate that BTN3A2 also is deeply involved in pAg-induced activation of Vγ9Vδ2 T cells (see also Figure 2) by regulating the appropriate routing, kinetics, and/or stability of BTN3A1.

Two mechanisms have been proposed to explain the interactions between BTN3A1 and pAgs and how these interactions are sensed by Vγ9Vδ2 T cells. Reports about how BTN3A proteins interact with pAgs are very conflicting and still represent an unsolved and intriguing question. The first mechanism postulates that pAgs are presented to Vγ9Vδ2 T cells *via* the membrane-distal IgV-like domain within the BTN3A1 ectodomain (57, 58). This model is reminiscent of the classical Ag-presentation model and implies that pAgs are made available in the extracellular space from endogenous or exogenous sources. However, following reports have demonstrated that pAgs interact directly with the intracellular B30.2 domain and failed to detect any association with the extracellular domains of BTN3A1 nor with the Vγ9Vδ2 TCRs (59–63).

The other mechanism is an inside-out mechanism initiated by interactions of the intracellular B30.2 domain with pAgs (59–63). Opposite to the antigen-presenting model, the allosteric model implies that signaling is operated by endogenous pAgs or exogenous pAgs after internalization from external sources. Within cells, pAgs are discriminated from non-antigenic small molecules because the former only may induce the conformational switch of the intracellular B30.2 domain (64). These changes determine the structural reorganization of BTN3A1 dimers on the cell surface which adopt a V-shaped conformation which is avidly recognized by Vγ9Vδ2 T cells. The inside-out signaling can be mimicked by agonistic (20.1) or antagonistic (103.2) antibodies which can induce or block the active conformation of BTN3A dimers on the cell surface (65) (Figure 2). It is still unclear how conformational changes of the intracellular B30.2 domain are transmitted to the cell surface. The juxtamembrane domain located close to the B30.2 domain has recently been reported to play an important role in the inside-out signal propagation (66, 67). The recruitment of other proteins like RhoB and periplakin has been proposed to participate to the structural reorganization of BTN3A1 dimers on the cell surface (61, 68). More recently, BTN3A2 also has been reported to be involved in the induction of active BTN3A1 conformation (56) (Figure 2). However, existing data require a cautious interpretation since in most cases they have been obtained using recombinant proteins and/or immobilized Vγ9Vδ2 TCRs. These experimental conditions do not recapitulate the dynamic situation going on under physiological or pathological conditions when much lower amounts of pAgs, binding proteins, and Vγ9Vδ2 TCRs are available. Likewise, some unsolved issues remain regarding the conformational switch induced by agonistic or antagonistic anti-BTN3A1 mAbs that may not fully mimic the conformational switch induced by pAgs, at least in some experimental models like the murine Vγ9Vγ2 TCR-transfectants reported by Starick et al (69). This variegate scenario is the propellant of an exciting debate about how BTN3A proteins interact with pAgs and stimulate Vγ9Vδ2 T cells.

Both the antigen presentation and the allosteric model implies pAg transportation across the cell membrane: the former implies that pAgs are made available in the extracellular space from endogenous sources; the latter that exogenous pAgs are internalized from external sources. BTN3A molecules are devoid of the capacity to transport pAgs across the membrane suggesting that interactions with other transporters are needed. The existence of an inside-out transporter was anticipated by De Libero and coworkers to support the antigen presentation model. Interestingly, these Authors have hypothesized the existence of a dedicated IPP transporter supervening only when there is an excessive IPP accumulation within APCs, but not when IPP is provided exogenously (58).

Based on these data, we have hypothesized that ABCA1, apoA-I, and BTN3A1 cooperate in the extracellular IPP release from ZA-treated cells. We believe that, whatever the mechanisms responsible for the conformational switch driven by intracellular IPP and/or agonistic/antagonistic anti-BTN3A1 mAb, it is mainly the active BTN3A1 conformation to participate to the ménage à trois and to facilitate extracellular IPP release in cooperation with ABCA1 and apoA-I. Intracellular IPP accumulation is boosted by ZA stimulation and this is propaedeutic to the acquisition of the active conformation fuctionally confirmed by the excellent ability of ZA-treated DCs to activate Vγ9Vδ2 T cells.

## The Cooperation Between ABCA1, apoA-I and BTN3A1

After screening the expression and activity of a large number of plasma membrane-associated ATPases, ABC transporters involved in lipid efflux and phosphate transporters, we found that ABCA1 only is upregulated by ZA treatment in DCs. Interestingly, this upregulation is accompanied by the simultaneous increase of apoA-I and IPP in the SNs (31). The highly significant correlation between the release of extracellular IPP and the expression of ABCA1 in many different cell types prompted us to further investigated the role of ABCA1/apoA-I in IPP efflux. The 3D structure of human ABCA1 has not yet been solved; the only available data indicate that apoA-I binds the extracellular amino-terminal domain of ABCA1 (52). Of note, we found that, in ZA-treated DCs, IPP binds to the same domain (31) (Figure 1). Only further proteo-lipidomic analysis of DC-derived SNs can provide the direct demonstration that IPP is physically associated to apoA-I. This will be a solid step toward the next challenge, i.e., understanding how extracellular IPP is presented to Vγ9Vδ2 T-cells (i.e., in soluble form, bound to Apo-AI, bound to BTN3A1). Our opinion is that ABCA1 extrudes IPP, but it cannot be an effective IPP-presenting molecule in a soluble form because it is a transmembrane protein unreleasable from viable cells. Whether the ABCA1/IPP/apoA-I complex can be released from apoptotic cells to provide activatory signals to Vγ9Vδ2 T cells is unknown. We have not determined the capacity of ZA-treated DCs to activate Vγ9Vδ2 T cells in cell-to-cell contact experiments after *Abca1* and/or *Btn3a1* silencing or knock-out. Additional experiments are needed to determine whether ABCA1 is just a safety valve supervening when potentially dangerous intracellular IPP concentrations are reached in DCs or whether ABCA1 is directly involved in pAg presentation.

As far as BTN3A1 is concerned, it appears to play the “third actor role” in the ABCA1/apoA-I/BTN3A1 ménage à trois (Figure 2). Contrarily to apoA-I, cross-linking experiments demonstrate that BTN3A1 does not bind to apoA-I, but co-immunoprecipitation and proximity-ligation assays indicate that BTN3A1 is physically associated with ABCA1 in DCs. It is really intriguing that, among all the possible partners available, BTN3A1 is physically and functionally associated with ABCA1 which extrudes IPP and whose expression is upregulated by IPP.

Zoledronic acid-treated *Abca1*-silenced DCs have a significant reduction in the ability to release extracellular IPP, whereas ZA-treated *Btn3A1-*silenced DCs are only marginally affected. However, when both genes are silenced in *Btn3a1/Abca1-*double silenced DCs, a statistically significant reduction in extracellular IPP release is observed compared with ZA-treated *Abca1*-silenced DCs (31).

We are aware that siRNA determine a partial and transient downregulation of BTN3A1 that can be different in different cell types. So far, data generated in our lab are sufficient to conclude that BTN3A1 participates to the ménage à trois facilitating extracellular IPP release by ABCA1 and apoA-I in ZA-treated DCs. A comparison between single and double *Abca1* and *Btn3a1* permanent knock-out cells could provide a more definitive conclusion about BTN3A1 involvement in IPP efflux.

Very recently, it has been reported that BTN3A2 also is required for optimal BTN3A1-mediated activation of Vγ9Vδ T cells (56). The interaction between these isoforms regulates the appropriate routing, kinetics, and stability of BTN3A1. Thus, we have envisaged an hypothetical scenario in which both BTN3A1 and BTN3A2 collaborate with ABCA1 and apoA-I to induce extracellular IPP release (Figure 2).

It is currently unknown whether the physical interaction between ABCA1 and BTN3A1 is a late event arising after that IPP-induced conformational changes have occurred or whether this is an early event contributing with IPP to the induction of BTN3A1 conformational changes. Since no physical interactions are detected between ABCA1 and BTN3A1 in the absence of ZA stimulation, and silencing *Abca1, Btn3a1*, or both genes, has no effect on extracellular IPP release in untreated DCs (31), the IPP/ABCA1/apoA-I/BTN3A1 cross-talk is likely initiated only after that supra-physiological IPP concentrations has been induced by ZA treatment. As of today, we cannot exclude that BTN3A1 interacts with other proteins, including BTN3A2 or other ABC transporters, to promote extracellular IPP release. Highly conserved and ubiquitous proteins like BTN3A1 are often part of multiprotein complexes where they may exert functions of adaptors, scaffold proteins or allosteric modulators of their interactors. Investigation of this putative BTN3A1 role is still in its infancy, but it could unravel very interesting and unexpected discoveries. Only an in-depth interactome study of BTN3A1 may identify other interactors involved in IPP efflux. Crystallography studies of BTN3A1-interactors complexes will provide additional information on the putative domains involved in IPP binding and subsequent IPP delivery to the interactors. Functional assays investigating IPP efflux, after selectively silencing the putative interactors, will shed light on the hierarchical function of each molecule in the process.

## Intracellular Signaling Involved in ABCA1/apoA-I Upregulation

Intracellular IPP binding to the B30.2 domain to induce BTN3A1 conformational changes and the concurrent upregulation of apoA-I and ABCA1 appear as a nicely coordinated process. 500 pM IPP, which is in the range of intracellular concentrations detected in ZA-treated DCs, is sufficient to activate the liver X receptor α (LXRα) and promote LXRα-induced transcription of *Abca1* and *apoA-I* (31). Putative ligands of LXRα in macrophages include several isoprenoid compounds, such as retinoic acid (70), astaxanthin (71), allyl-cysteine (72), or zerumbone (73). In DCs, however, the effect of IPP is highly specific and neither exogenously added FPP or GGPP induce LXRα activation (31). The different chain length and tridimensional conformation may account for the different ability to induce LXRα activation. Moreover, ZA decreases intracellular FPP and GGPP concentrations to sub-picomolar values (74–76) which are insufficient to induce LXRα activation (31).

These data also point out how different can be the transcriptional regulation of Abca1/apoA-I and lipid metabolism in immune cells. In DCs, *Abca1* expression is mainly regulated by LXRα and IPP-induced ABCA1 upregulation is finalized to extrude IPP in cooperation with apoA-I and BTN3A1; moreover, DCs express very low levels of LXRβ (77) which remains unmodulated by ZA (31). By contrast, *Abca1* expression in T cells is governed mainly by LXRβ and ABCA1 upregulation induces cholesterol depletion and impairs T-cell functions (78). It is currently unknown whether a similar ménage à trois occurs in T cells as a consequence of LXRβ-induced ABCA1 activation.

A second IPP-independent mechanism by which ZA upregulates ABCA1/apoA-I complex in DCs is the intracellular shortage of FPP generated by FPPS inhibition, decreased Ras prenylation (74) and decreased activity of the Ras-dependent PI3K/Akt/mTOR pathway (79) which constitutively inhibits LXRα activation (31, 80). PI3K/Akt-activity also reduces the amount of surface ABCA1, likely interfering with recycling mechanisms (81). ERK1/2, other Ras-downstream effectors (73), negatively regulate ABCA1 expression in macrophages (82). Although not yet explored in DCs, also this Ras-dependent pathway may be involved in ABCA1 expression and ABCA1-dependent IPP efflux.

## Conclusion

In conclusion, the ménage à trois between apoA-I, ABCA1, and BTN3A1 in ZA-treated DCs is finalized to extrude very efficiently intracellular IPP after that supra-physiological concentrations have been reached as a consequence of a deranged Mev pathway. The relationships between these partners are hierarchically not equivalent because ABCA1 and apoA-I are physically associated (as expected), ApoA-I and BTN3A1 are physically associated, whereas BTN3A1 and apoA-I are not physically associated. IPP binds to ABCA1, BTN3A1, and apoA-I, further promoting interactions between these molecules. We speculate that the aim of this mènage à trois is twofold: the first is to extend the range of immune regulation also to Vγ9Vδ2 T cells which are not in close proximity to pAg-presenting cells. Under this perspective, IPP efflux can be included in the number of the damage-associated molecular patterns, as microbial pAgs can be considered part of pathogen-associated molecular patterns, i.e., highly conserved pathways that trigger and sustain immune activity in response to danger signals (83). The second is to protect pAg-presenting cells from apoptosis due to intracellular ApppI accumulation. It is reasonable that immune cells have developed pleiotropic mechanisms to protect themselves from IPP accumulation. A redundancy of mechanisms controlling the IPP efflux may ensure that the immune activation of Vγ9Vδ2 T cells operated by pAg-presenting cells is not prematurely terminated.

## Author Contributions

All authors have made substantial contributions to text and figures and have approved the manuscript for submission.

## Conflict of Interest Statement

The authors declare that the research was conducted in the absence of any commercial or financial relationships that could be construed as a potential conflict of interest.
